# Role of Ras in regulation of intestinal epithelial cell homeostasis and crosstalk with Wnt signaling

**DOI:** 10.1371/journal.pone.0256774

**Published:** 2021-08-26

**Authors:** Takenori Kotani, Noriko Ihara, Saki Okamoto, Jajar Setiawan, Tasuku Konno, Yasuyuki Saito, Yoji Murata, Takashi Matozaki

**Affiliations:** 1 Division of Molecular and Cellular Signaling, Department of Biochemistry and Molecular Biology, Kobe University Graduate School of Medicine, Kobe, Japan; 2 Department of Physiology, Faculty of Medicine, Public Health, and Nursing, Universitas Gadjah Mada, Yogyakarta, Indonesia; Kindai University: Kinki Daigaku, JAPAN

## Abstract

Cross talk between different signaling pathways is thought to be important for regulation of homeostasis of, as well as oncogenesis of, the intestinal epithelium. Expression of an active form of K-Ras specifically in intestinal epithelial cells (IECs) of mice (IEC-RasDA mice) resulted in the development of hyperplasia in the small intestine and colon of mice. IEC-RasDA mice also manifested the increased proliferation of IECs. In addition, the number of goblet cells markedly increased, while that of Paneth cells decreased in IEC-RasDA mice. Development of intestinal organoids was markedly enhanced for IEC-RasDA mice compared with control mice. Whereas, the expression of Wnt target genes was significantly reduced in the in intestinal crypts from IEC-RasDA mice compared with that apparent for the control. Our results thus suggest that K-Ras promotes the proliferation of IECs as well as generation of goblet cells. By contrast, Ras counter-regulates the Wnt signaling and thereby contribute to the proper regulation of intestinal epithelial cell homeostasis.

## Introduction

Intestinal stem cells (ISCs), which reside at the base of intestinal crypts, maintain renewal of intestinal epithelial cells (IECs) by generating proliferating progeny, known as transient amplifying (TA) cells [1, 2]. Above the stem cell niche in the crypt, TA cells divide actively and differentiate into the various IECs such as absorptive enterocytes, mucin-producing goblet cells, antimicrobial peptide–producing Paneth cells, and peptide hormone–secreting enteroendocrine cells. The best characterized signaling as a positive regulator for maintaining ISCs is the Wnt–β-catenin signaling [3, 4]. Wnt ligands, such as Wnt3, are predominantly secreted by Paneth cells and activates the Wnt–β-catenin signaling in IECs [3]. The Wnt–β-catenin signaling normally promotes the proliferation of ISCs or TA cells and the maturation of Paneth cells [4, 5]. In contrast, aberrant activation of Wnt–β-catenin signaling in IECs likely contributes to tumorigenesis in the intestine. Indeed, ablation by genetic mutation or deletion of Apc, which is a negative regulator for the Wnt–β-catenin signaling, causes intestinal tumorigenesis in mouse and human [6].

Epidermal growth factor (EGF) also plays important roles in the proliferation of ISCs or TA cells [7]. EGF activates EGF receptor tyrosine kinase and stimulates the Ras-Erk (extracellular signal-regulated kinase) signaling. The Ras family of proteins comprises K-Ras, H-Ras, and N-Ras [8], and mutation of *K-ras* gene are found in ~40% of colorectal cancer [9]. Interestingly, a recent study demonstrated that an inhibitor for the secretion of Wnt ligands promoted the activation of Erk and converted ISCs into TA cells at the base of crypts [10]. This finding suggests that Wnt ligands suppress the Erk activity to maintain a pool of ISCs at the crypt base. Conversely, inhibition of Ras by ablation of Shp2, a protein tyrosine phosphatase that is essential for activation by growth factors of Ras [11, 12], increased the expression of stem-cell–associated genes as well as Wnt target genes in the intestine [13]. In addition, ablation of Erk1/2 also activates the Wnt–β-catenin signaling pathway in IECs [14]. Thus, the Ras-Erk signaling likely suppresses the Wnt signaling in IECs, the physiological role of such regulation by Ras for IEC homeostasis remains poorly understood. In this study, we thus generated the IEC-RasDA mice, in which K-Ras was specifically activated in IECs, to re-evaluate the role of Ras in regulation of IEC homeostasis, as well as of Wnt signaling, in mice and the organoid culture.

## Materials and methods

### Ethics statement

This study was approved by the Institutional Animal Care and Use Committee of Kobe University (permit numbers P170707 and P190902), and animal experiments were performed according to Kobe University Animal Experimentation Regulations. Mice were maintained at the Institute for Experimental Animals at Kobe University Graduate School of Medicine under specific pathogen–free conditions. Mice were housed in 12 h light-dark cycle and given normal chow (CLEA Rodent diet CE-2, CLEA Japan, Tokyo, Japan) and water ad libitum. Mice were checked daily and cages were cleaned weekly. Mice were sacrificed by cervical dislocation under anesthesia with isoflurane when samples were prepared. All efforts were made to minimize suffering.

### Mice

K-ras^*LSL-Kras G12D/+*^ mice [15], *Villin-Cre* mice [16], and *Lgr5-Gfp-CreERT2* (Lgr5-GFP) mice [17] were obtained from Jackson Laboratory (Bar Harbor, ME). K-ras^*LSL-Kras G12D/+*^ mice were crossed with *Villin-Cre* mice to obtain K-ras^*LSL-Kras G12D/+*^;*Villin-Cre* (IEC-RasDA) and K-ras^*LSL-Kras G12D/+*^ (control) mice. These mice were also crossed with *Lgr5-Gfp-CreERT2* mice to obtain K-ras^*LSL-Kras G12D/+*^;*Villin-Cre*;*Lgr5-Gfp-CreERT2* (IEC-RasDA/Lgr5-GFP) and K-ras^*LSL-Kras G12D/+*^;*Lgr5-Gfp-CreERT2* (control/Lgr5-GFP) mice.

### Antibodies and reagents

Rabbit polyclonal antibodies (pAbs) to Ki67 were obtained from Acris (Herford, Germany). Rabbit pAbs to Muc2 were obtained from Santa Cruz Biotechnology (Santa Cruz, CA). Rabbit pAbs to lysozyme were obtained from Dako (Glostrup, Denmark). Rabbit monoclonal antibody (mAb) to Erk1/2 and rabbit pAbs to phosphorylated Erk1/2 (Thr202/Tyr204) were obtained from Cell Signaling Technology (Beverly, MA). A mouse mAb to β-catenin was obtained from BD Biosciences (San Diego, CA). Horseradish peroxidase–conjugated secondary antibodies for immunoblot analysis and Cy3-conjugated secondary antibodies for immunofluorescence analysis were obtained from Jackson ImmunoResearch (West Grove, PA). Alexa Fluor 488-conjugated secondary antibodies for immunofluorescence analysis were obtained from ThermoFisher (Waltham, MA). Mayer’s hemalum solution was obtained from Merck KGaA (Darmstadt, Germany). Eosin was obtained from Wako (Osaka, Japan). 4′,6-diamidino-2-phenylindole (DAPI) was obtained from Nacalai Tesque (Kyoto, Japan).

### Immunoblot analysis

The ileum or colon from mice was homogenized with lysis buffer [20 mM Tris-HCl (pH 7.5), 150 mM NaCl, 2 mM EDTA, 1% Nonidet P-40, 1% sodium deoxycholate, 0.1% sodium dodecyl sulfate, 50 mM NaF, 1 mM sodium vanadate, and 1% protease inhibitor cocktail (Nacalai Tesque)]. The lysates were subjected to immunoblot analysis as previously described [18–20].

### Histology and immunohistofluorescence analysis

The ileum and colon from mice were incubated in PBS containing 4% paraformaldehyde for 3 h at room temperature. After fixation, the tissue was incubated in PBS containing 30% (w/v) sucrose for cryoprotection. The tissue was then frozen in optimal cutting temperature compound (Sakura, Tokyo, Japan). Frozen sections with a thickness of 5 μm were prepared with a cryostat. For histological analysis, sections were stained with hematoxylin-eosin. For immunohistofluorescence analysis, sections were stained with antibodies as described previously [18, 21, 22]. Sections were also stained with DAPI to detect nuclei. Images were obtained with a fluorescence microscope (BX51; Olympus, Tokyo, Japan). The fluorescence intensity was measured with the use of ImageJ software (NIH).

### Isolation of mouse intestinal crypts and quantitative real-time PCR analysis

Isolation of total RNA from mouse ileal or colonic crypts were performed as described previously [20, 23], with minor modifications. For isolation of mouse ileal crypts, the mouse ileum was incubated for 30 min in PBS containing 5 mM EDTA at 4°C. For isolation of mouse colonic crypts, the mouse colon was incubated for 30 min in PBS containing 5 mM EDTA at 4°C and then incubated for 30 min in Dulbecco’s modified Eagle’s medium–F12 (Invitrogen, Carlsbad, CA) containing 10 mM HEPES (Invitrogen) and collagenase type IV (500 U/ml) (Worthington Biochemical, Lakewood, NJ) at 37°C. Total RNA from ileal or colonic crypts was then isolated by using Sepasol RNA I (Nacalai Tesque) and RNeasy Mini Kit (Qiagen, Hilden, Germany). Reverse transcription and quantitative real-time PCR analysis were performed as described previously [20, 23]. The data were normalized by the amount of hypoxanthine-guanine phosphoribosyltransferase 1 (Hprt1) mRNA. Primer sequences (forward and reverse, respectively) were as follows: Hprt1, 5’-CAGTCCCAGCGTCGTGATTA-3’ and 5’-GGCCTCCCATCTCCTTCATG-3’; Lgr5, 5’-ACCCGCCAGTCTCCTACATC-3’ and 5’-GCATCTAGGCGCAGGGATTG-3’; Ascl2, 5’-CTACTCGTCGGAGGAAAG-3’ and 5’-ACTAGACAGCATGGGTAAG-3’; Axin2, 5’-GGACTGGGGAGCCTAAAGGT-3’ and 5’-AAGGAGGGACTCCATCTACGC-3’; cyclin D1, 5’-CAGACGTTCAGAACCAGATTC-3’ and 5’-CCCTCCAATAGCAGCGAAAAC-3’.

### Intestinal organoid culture

Intestinal organoid culture was performed as previously described [7, 21, 24] but with a slight modification. In brief, ileal crypts were isolated as described above, mixed with Matrigel (BD Biosciences), and transferred to 48-well plates. Advanced Dulbecco’s modified Eagle’s medium–F12 (Invitrogen) supplemented with 10% R-spondin1–Fc–conditioned medium, epidermal growth factor (50 ng/ml) (Peprotech, Rocky Hill, NJ), Noggin (100 ng/ml) (Peprotech), 10 mM HEPES, 1× GlutaMAX (Invitrogen), 1× B27 (Invitrogen), 1× N2 (Invitrogen), 1.25 mM *N*-acetylcysteine (Sigma-Aldrich), and penicillin-streptomycin (100 U/ml) was added in each well after polymerization of the Matrigel. The intestinal organoids were cultured in an incubator at 37°C with 5% CO_2_. Images of intestinal organoids were obtained with an CKX53 microscope (Olympus). The area of intestinal organoid was measured with the use of ImageJ software (NIH) and the number of budding of each intestinal organoid was visually counted.

### Statistical analysis

Data are presented as means ± s.e. and were analyzed with 2-tailed Student’s *t* test with the use of GraphPad Prism software version 6.0 (GraphPad, San Diego, CA). A *P* value of less than 0.05 was considered statistically significant.

## Results

### Hyperplasia of the intestinal epithelium in mice expressing an activated form of Ras specifically in IECs

**Fig 1:**
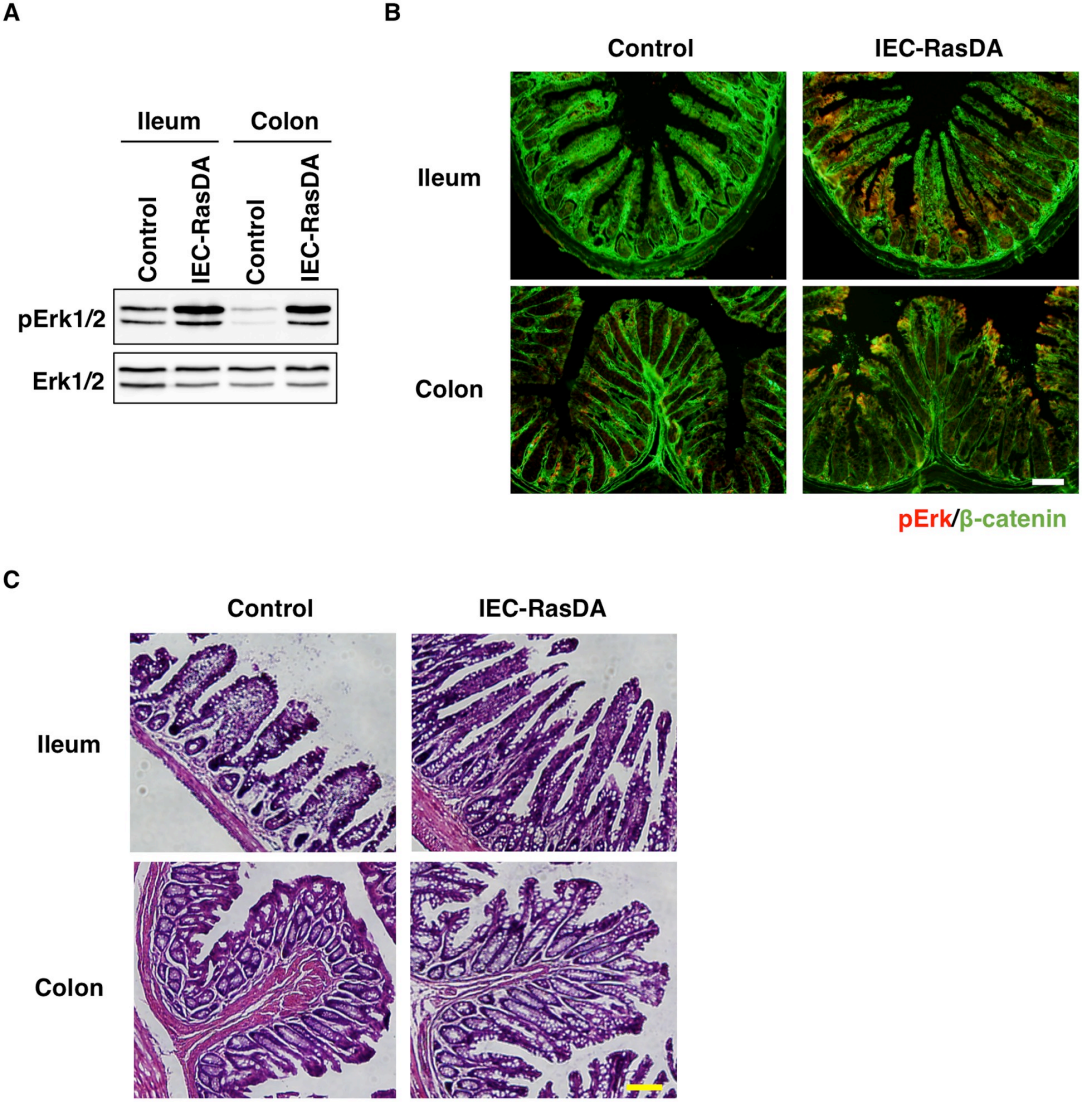
Hyperplasia of the intestinal epithelium in IEC-RasDA mice. (**A**) Lysates of the ileum and colon from control or IEC-RasDA mice at 12-week-old were subjected to immunoblot analysis with antibodies to phosphorylated (p) or total forms of Erk1/2. (**B**) Frozen sections of the ileum and colon from control or IEC-RasDA mice at 10-week-old were subjected to immunohistofluorescence analysis with antibodies to pErk1/2 (red) and to β-catenin (green). Scale bar, 100 μm. (**C**) Hematoxylin-eosin staining of frozen sections of the ileum and colon from control or IEC-RasDA mice at 12-week-old. Scale bar, 100 μm.

To investigate the role of Ras in the regulation of intestinal epithelial homeostasis, we generated mice (IEC-RasDA mice) expressing a dominant-activated form of Ras specifically in IECs by crossing of Kras^*LSL-Kras G12D/+*^ mice [15], which have a gene for an activated form of K-Ras (K-Ras^G12D^), with *Villin-Cre* mice [16]. Such crossing removes translational termination sequence (loxP-STOP-loxP) by Cre-mediated recombination and leads expression of the K-Ras^G12D^ gene in an IEC-specific manner. Immunoblot analysis showed that the phosphorylation level of Erk1/2 (extracellular signal-regulated kinase 1/2), which reflects the Ras activity [25], was markedly increased in the ileum and colon of IEC-RasDA mice compared with that apparent for the control mice (Fig 1A). Immunohistofluorescence analysis also revealed more prominent staining for phosphorylated Erk1/2 in IECs from ileal or colonic mucosa of IEC-RasDA mice compared with those of control mice (Fig 1B). These results indicated that K-Ras and its downstream signaling pathway were indeed activated specifically in IECs of IEC-RasDA mice.

Histological examination revealed that epithelial hyperplasia was pronounced in the ileum and colon of IEC-RasDA mice compared with control mice (Fig 1C). In addition, serrated glandular morphology was observed in the ileal and colonic epithelium of IEC-RasDA mice (Fig 1C). These results thus suggested that activation of K-Ras promotes the proliferation of IECs in the small intestine and colon as previously described [26].

### Increased proliferation of crypt IECs and increased number of goblet cells as well as decreased number of Paneth cells in in IEC-RasDA mice

**Fig 2:**
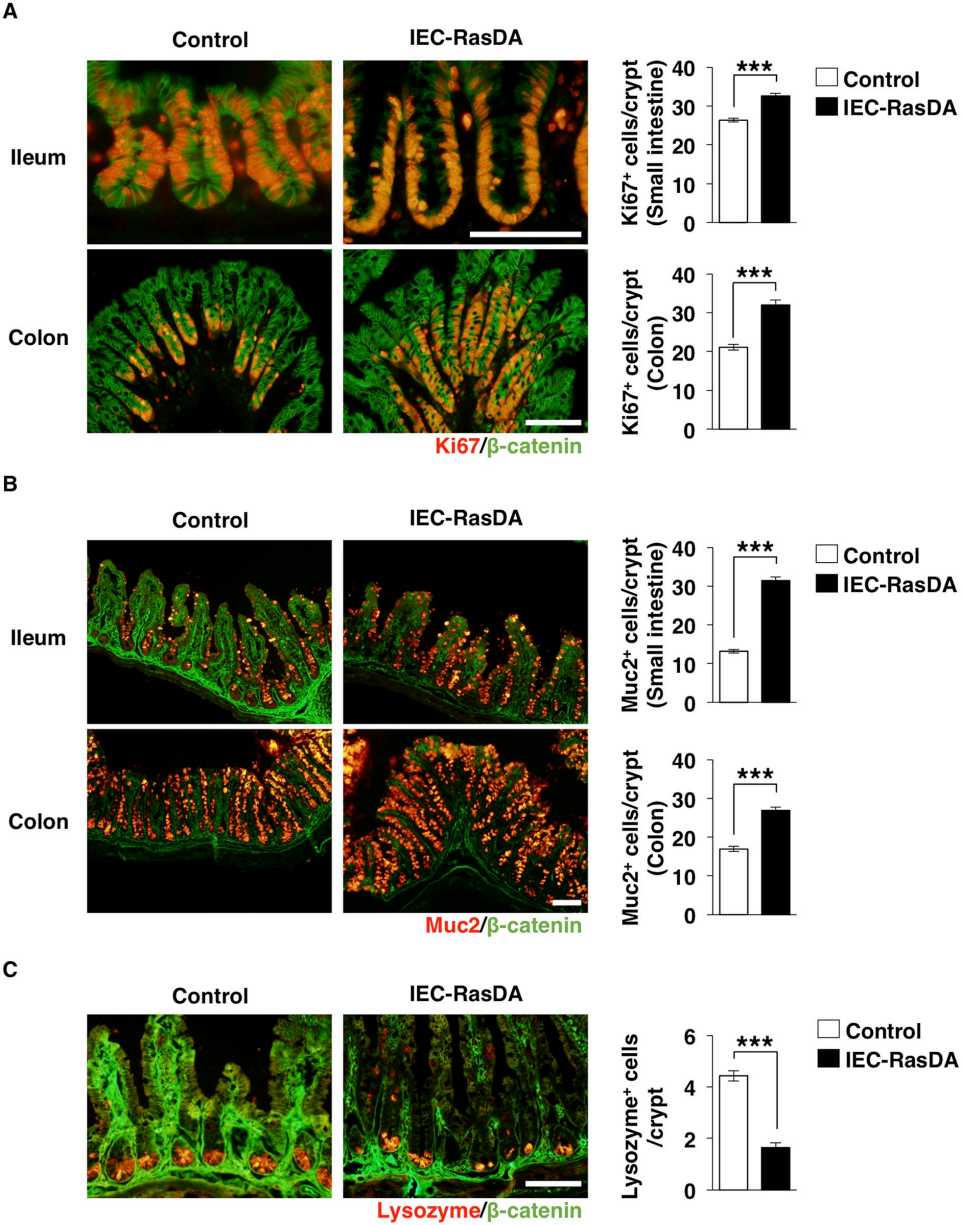
Proliferation and differentiation of IECs in IEC-RasDA mice. (**A**) Frozen sections of the ileum and colon from control or IEC-RasDA mice were subjected to immunohistofluorescence analysis with antibodies to Ki67 (red) and to β-catenin (green). Scale bar, 100 μm. The number of Ki67-positive cells per crypt is also shown in right panels. (**B**) Frozen sections of the ileum and colon from control or IEC-RasDA mice were subjected to immunohistofluorescence analysis with antibodies to mucin 2 (Muc2) (red) and to β-catenin (green). Scale bar, 100 μm. The number of Muc2-positive cells per crypt is also shown in right panels. (**C**) Frozen sections of the ileum from control or IEC-RasDA mice were subjected to immunohistofluorescence analysis with antibodies to lysozyme (red) and to β-catenin (green). Scale bar, 100 μm. The number of lysozyme-positive cells per crypt is also shown in the right panel. Quantitative data are means ± s.e. for 90 crypts from three control and three IEC-RasDA mice at 10- to 12-week-old. ***P < 0.001 (Student’s *t* test).

We thus analyzed the proliferation of IECs in IEC-RasDA mice by immunostaining for Ki67, which is known as a marker of cell proliferation [27]. We found that the number of Ki67-positive cells was markedly increased in crypts of the ileum and colon of IEC-RasDA mice (Fig 2A). These results suggested that Ras indeed promotes the proliferation of crypt IECs such as TA cells.

We next investigated the role of IEC-specific activation of K-Ras in the differentiation of IECs. The number of goblet cells, which were mucin 2-positive cells, was markedly increased in the ileum and colon of IEC-RasDA mice (Fig 2B). In contrast, we found that the number of Paneth cells, which were lysozyme-positive cells [28], was significantly reduced in the ileum of IEC-RasDA mice (Fig 2C). These results thus suggested that K-Ras positively regulates generation of goblet cells but it negatively regulates that of Paneth cells.

### Promotion of the development of intestinal organoids from IEC-RasDA mice

**Fig 3:**
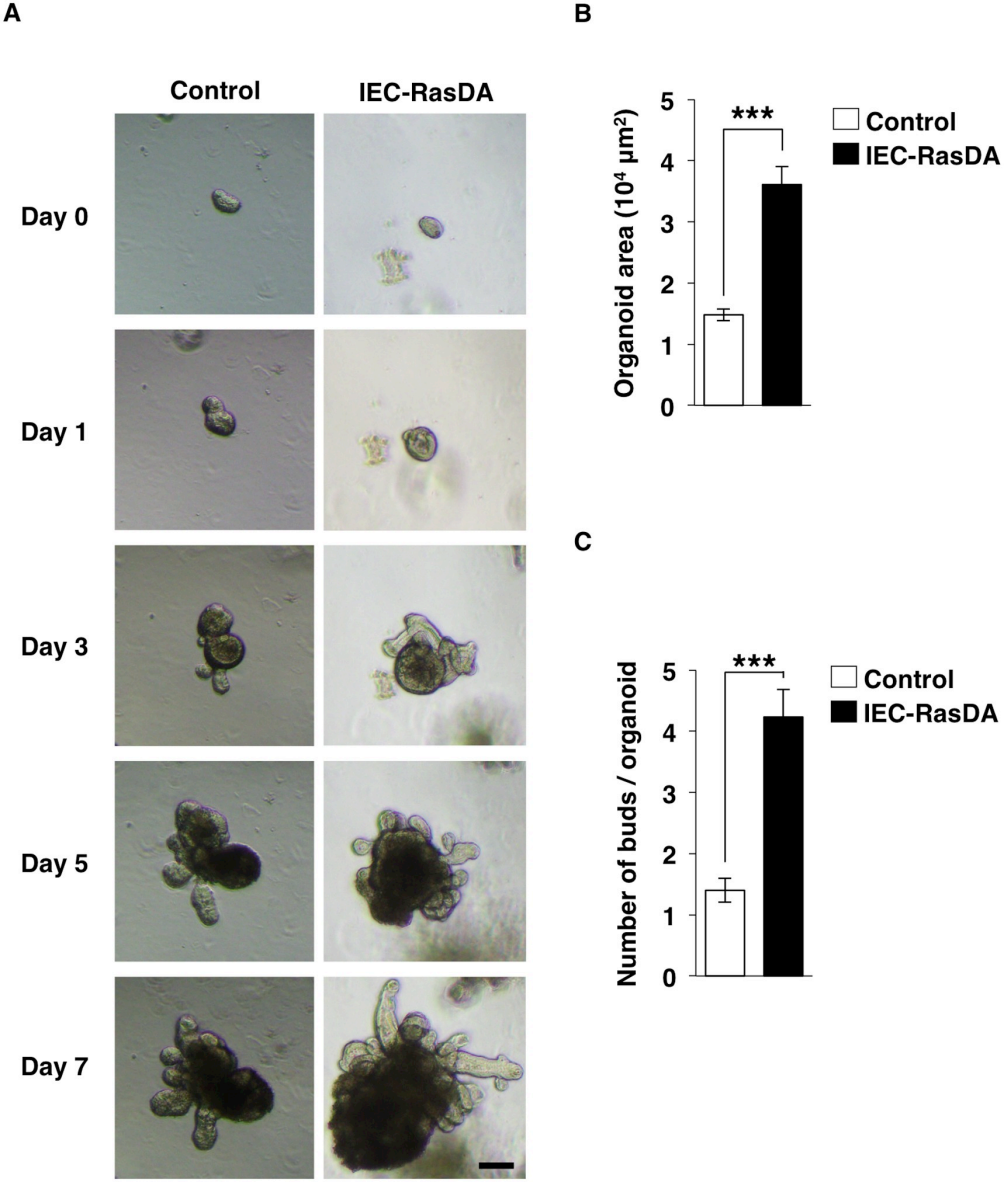
Promotion of the development of intestinal organoids by K-Ras activation. (**A**) Images of intestinal organoids derived from the ileum of 12-week-old control or IEC-RasDA mice at the indicated times after cell plating. Scale bar, 100 μm. (**B**) Areas of intestinal organoids at 3 days after cell plating. (**C**) The number of buds per intestinal organoid at 3 days after cell plating. Data are means ± s.e. for a total of 30 organoids from control or IEC-RasDA mice at 12-week-old. ***P < 0.001 (Student’s *t* test).

The intestinal organoids prepared from isolated intestinal crypts are thought to mimic the proliferation and differentiation of IECs *in vivo* [7, 29]. To clarify further the role of K-Ras in IECs, we examined the development of intestinal organoids prepared from control and IEC-RasDA mice. Isolated crypts from control or IEC-RasDA mice gradually developed into intestinal organoids with budding crypts (Fig 3A). At 3days after cell seeding, the surface area of K-Ras-activated intestinal organoids had become larger than that of control organoids (Fig 3A and 3B). The number of buds was also increased for the organoids from IEC-RasDA mice (Fig 3A and 3C). These results thus suggested that K-Ras activation in IECs promotes the development of intestinal organoids. Furthermore, the phenotypes of IECs in IEC-RasDA mice are likely caused by cell-autonomous effects of K-Ras-activation.

### Down-regulation of Wnt-target genes in IECs from IEC-RasDA mice

**Fig 4:**
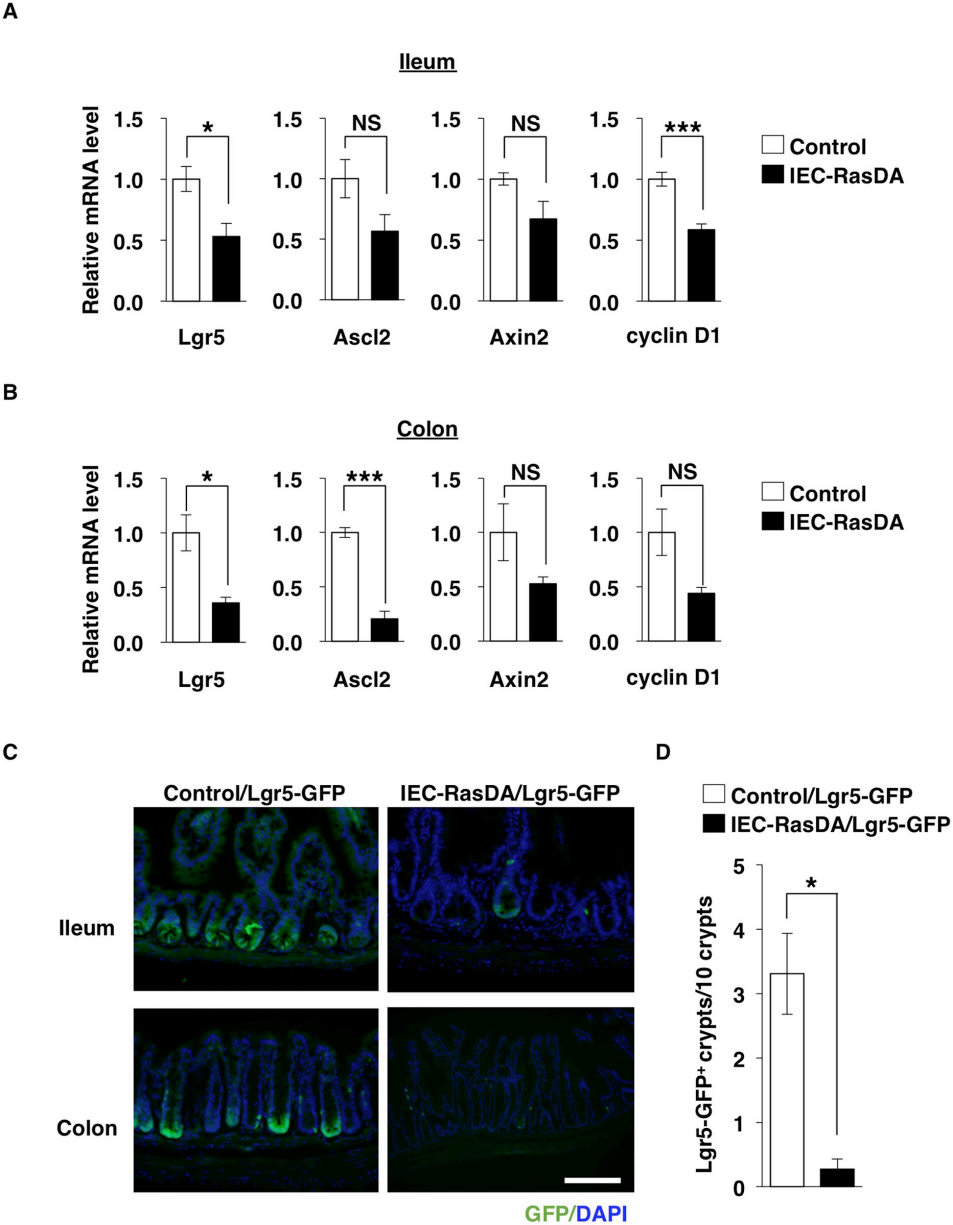
Down-regulation of Wnt-target genes in crypts from IEC-RasDA mice. (**A, B**) Expression of Lgr5, Ascl2, Axin2, and cyclin D1 mRNAs in crypts isolated from the ileum (**A**) or the colon (**B**) of control or IEC-RasDA mice at 11- to 16-week-old. Data are means ± s.e. from three separate experiments. *P < 0.05; ***P < 0.001; NS, not significant (Student’s *t* test). (**C**) Frozen sections of the ileum and colon from control/Lgr5-GFP and IEC-RasDA/Lgr5-GFP mice at 10- to 11-week-old were examined for GFP fluorescence (green). These sections were stained with DAPI (blue) to detect nuclei. Scale bar, 100 μm. (**D**) The number of Lgr5-GFP–positive ileal crypts per 10 crypts. Data are means ± s.e. from three separate experiments (346 crypts from three control/Lgr5-GFP mice and 277 crypts from three IEC-RasDA /Lgr5-GFP mice were analyzed). *P < 0.05 (Student’s *t* test).

Leucine-rich repeat–containing G protein–coupled receptor 5 (Lgr5)–positive ISCs are thought to self-renew and to generate TA cells in the intestinal epithelium [1, 17]. Given that ablation of Shp2, a protein tyrosine phosphatase which is essential for activation by growth factors of Ras, up-regulates the expression of stem-cell–associated genes and Wnt target genes in the small intestine [13], we focused on the Wnt–β-catenin signaling in IECs from IEC-RasDA mice. Although the activation of Wnt–β-catenin signaling translocates β-catenin to the nucleus [30], we could not detect the difference of β-catenin localization in IECs between IEC-RasDA mice and control mice by immunohistofluorescence analysis (S1 Fig). These results may be due to the too low fluorescence intensity of β-catenin in the nucleus. In contrast, RT-PCR analysis revealed that the expression of Lgr5 [31], a marker of ISC, was markedly reduced in crypts isolated from the ileum and colon from IEC-RasDA mice compared with those isolated from control mice (Fig 4A and 4B). In addition, expression of the Wnt–β-catenin target genes, such as *Ascl2*, *Axin2*, and *cyclin D1*, was significantly decreased or tended to be decreased in crypts isolated from the ileum or colon of IEC-RasDA mice compared with those isolated from control mice (Fig 4A and 4B). To further investigate the effect of K-Ras activation on the expression of Lgr5 in ISCs we crossed either control or IEC-RasDA mice with *Lgr5-Gfp-CreERT2* (Lgr5-GFP) mice, which express GFP under the control of the Lgr5 gene promoter [17] in ISCs, and examined the number of GFP-positive crypts in the ileum and colon. The number of GFP-positive crypts in either ileal or colonic mucosa was markedly reduced in IEC-RasDA /Lgr5-GFP mice compared with control/Lgr5-GFP mice (Fig 4C and 4D). These results suggested that K-Ras activity likely regulates the expression of Lgr5 in ISCs of the crypt through suppressing the Wnt–β-catenin signaling pathway.

## Discussion

**Fig 5:**
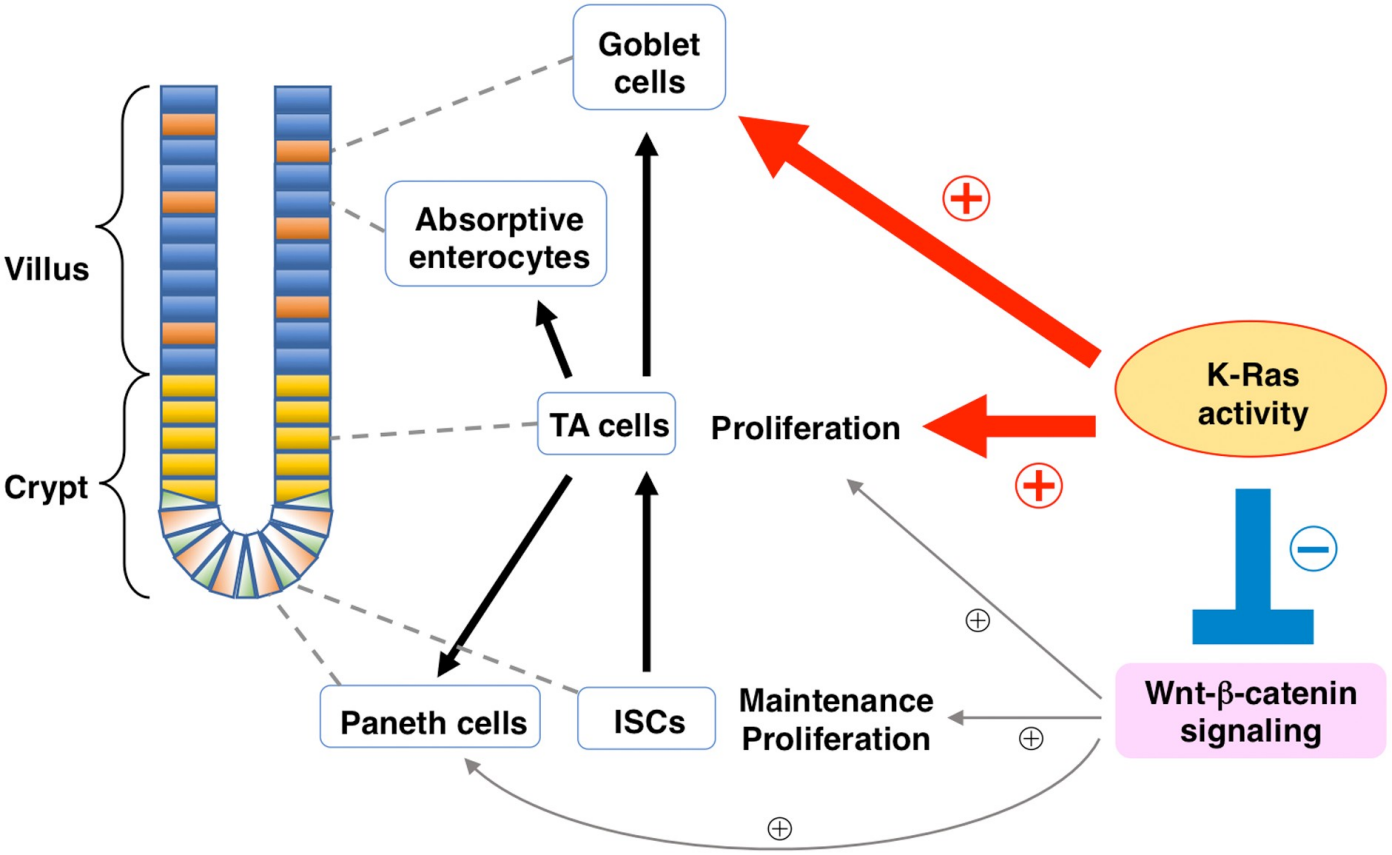
Regulation by K-Ras activity of IEC homeostasis. The Ras signaling pathway promotes both IEC proliferation and the differentiation of goblet cells. In contrast, the Ras activity primarily downregulates Wnt–β-catenin signaling and negatively regulates in the number of Paneth cells as well as of ISCs at the crypt base.

We have here shown that the proliferation of IECs is markedly increased in the intestinal epithelium of IEC-RasDA mice. Moreover, the development of intestinal organoids was promoted by K-Ras activation in IECs. Given that K-Ras is a downstream molecule of growth factor receptors such as EGF receptor, our results thus suggest that K-Ras plays an important role in promotion of the proliferation of IECs. We also found that the number of Muc2-positive mucus-secreting goblet cells was increased and that of lysozyme-positive Paneth cells was decreased in IEC-RasDA mice. Previously, we and others demonstrated that ablation of Shp2 specifically in IECs decreased the number of goblet cells and increased that of Paneth cells [13, 21]. Thus, the activity of K-Ras, a downstream molecule of Shp2, is likely essential for differentiation of these IECs from their progenitors (Fig 5).

The most notable finding in this study is that activation of K-Ras in IECs reduced the expression of Lgr5 and Wnt target genes in the crypts of ileum and colon. The decrease of Paneth cells, which secrete Wnt ligands, might be responsible for the reduced expression of these genes in the ileum. Whereas, the expression of these genes was also reduced in the colon of the IEC-RasDA mice even though Paneth cells are rarely present in the colon. These results suggested that the activation of K-Ras down-regulates Wnt–β-catenin signaling in IECs of intestinal crypts and thus negatively regulates in the number of Paneth cells as well as of Lgr5-positive ISCs at the crypt base (Fig 5). This notion is consistent with the previous observation that ablation of Shp2 up-regulates the expression of stem-cell–associated genes and Wnt target genes [13]. In addition, a recent study demonstrated that the ablation of Erk1/2 activated the Wnt–β-catenin signaling in IECs [14]. In contrast, inhibition of Wnt secretion promotes the activation of Erk and conversion of ISCs into TA cells at the base of crypts [10]. Given that the activity of Wnt–β-catenin signaling is strongest at the crypt base and it gradually decreases along the crypt-villus axis within the crypt [32], such gradient of the activity is likely important for both maintenance of ISCs at the crypt base and the proliferation of TA cells or mature IECs at the upper crypt, respectively. Our findings suggest that K-Ras activity opposes Wnt–β-catenin signaling and promotes TA cells or mature IEC proliferation at the upper region of the crypt. Clarification of the molecular basis for the counter-regulation by Ras of Wnt–β-catenin signaling will be required for further understanding of the regulatory mechanism of IEC homeostasis.

## Supporting information

S1 Fig Localization of β-catenin in IECs of control and IEC-RasDA mice. (**A**) Frozen sections of the ileum from control or IEC-RasDA mice were stained with antibodies to β-catenin (green) and DAPI (blue). Scale bar, 10 μm. Relative β-catenin fluorescence (green fluorescence) intensity in nucleus of IECs of the ileum is also shown in the right panel. Quantitative data are means ± s.e. for 60 IECs from control or IEC-RasDA mice. NS, not significant (Student’s *t* test). (**B**) Frozen sections of the colon from control or IEC-RasDA mice were stained with antibodies to β-catenin (green) and DAPI (blue). Scale bar, 10 μm. Relative β-catenin fluorescence (green fluorescence) intensity in nucleus of colonic epithelial cells is also shown in the right panel. Quantitative data are means ± s.e. for 60 colonic epithelial cells from control or IEC-RasDA mice. NS, not significant (Student’s *t* test).
(PDF)

S1 Raw images Uncropped immunoblot for Fig 1A. (PDF)

## References

[1] Barker N, Huch M, Kujala P, van de Wetering M, Snippert HJ, van Es JH, et al. Lgr5^+^ stem cells drive self-renewal in the stomach and build long-lived gastric units in vitro. Cell Stem Cell. 2010;6(1):25–36. doi: 10.1016/j.stem.2009.11.013 20085740

[2] Noah TK, Donahue B, Shroyer NF. Intestinal development and differentiation. Exp Cell Res. 2011;317(19):2702–10. doi: 10.1016/j.yexcr.2011.09.006 21978911 PMC3210330

[3] Sato T, van Es JH, Snippert HJ, Stange DE, Vries RG, van den Born M, et al. Paneth cells constitute the niche for Lgr5 stem cells in intestinal crypts. Nature. 2011;469(7330):415–8. doi: 10.1038/nature09637 21113151 PMC3547360

[4] Gregorieff A, Clevers H. Wnt signaling in the intestinal epithelium: from endoderm to cancer. Genes Dev. 2005;19(8):877–90. doi: 10.1101/gad.1295405 15833914

[5] Clevers H. The intestinal crypt, a prototype stem cell compartment. Cell. 2013;154(2):274–84. doi: 10.1016/j.cell.2013.07.004 23870119

[6] Fodde R, Smits R, Clevers H. APC, signal transduction and genetic instability in colorectal cancer. Nat Rev Cancer. 2001;1(1):55–67. doi: 10.1038/35094067 11900252

[7] Sato T, Vries RG, Snippert HJ, van de Wetering M, Barker N, Stange DE, et al. Single Lgr5 stem cells build crypt-villus structures in vitro without a mesenchymal niche. Nature. 2009;459(7244):262–5. doi: 10.1038/nature07935 19329995

[8] Takai Y, Sasaki T, Matozaki T. Small GTP-binding proteins. Physiol Rev. 2001;81(1):153–208. doi: 10.1152/physrev.2001.81.1.153 11152757

[9] De Roock W, Claes B, Bernasconi D, De Schutter J, Biesmans B, Fountzilas G, et al. Effects of KRAS, BRAF, NRAS, and PIK3CA mutations on the efficacy of cetuximab plus chemotherapy in chemotherapy-refractory metastatic colorectal cancer: a retrospective consortium analysis. Lancet Oncology. 2010;11(8):753–62. doi: 10.1016/S1470-2045(10)70130-3 20619739

[10] Kabiri Z, Greicius G, Zaribafzadeh H, Hemmerich A, Counter CM, Virshup DM. Wnt signaling suppresses MAPK-driven proliferation of intestinal stem cells. J of Clin Invest. 2018;128(9):3806–12. doi: 10.1172/JCI99325 30059017 PMC6118584

[11] Matozaki T, Murata Y, Saito Y, Okazawa H, Ohnishi H. Protein tyrosine phosphatase SHP-2: a proto-oncogene product that promotes Ras activation. Cancer Sci. 2009;100(10):1786–93. doi: 10.1111/j.1349-7006.2009.01257.x 19622105 PMC11158110

[12] Neel BG, Gu H, Pao L. The ’Shp’ing news: SH2 domain-containing tyrosine phosphatases in cell signaling. Trends Biochem Sci. 2003;28(6):284–93. doi: 10.1016/S0968-0004(03)00091-4 12826400

[13] Heuberger J, Kosel F, Qi J, Grossmann KS, Rajewsky K, Birchmeier W. Shp2/MAPK signaling controls goblet/paneth cell fate decisions in the intestine. Proc Natl Acad Sci U S A. 2014;111(9):3472–7. doi: 10.1073/pnas.1309342111 24550486 PMC3948231

[14] Wei GG, Gao N, Chen JW, Fan LL, Zeng ZY, Gao GL, et al. Erk and MAPK signaling is essential for intestinal development through Wnt pathway modulation. Development. 2020;147(17):dev185678. doi: 10.1242/dev.185678 32747435

[15] Jackson EL, Willis N, Mercer K, Bronson RT, Crowley D, Montoya R, et al. Analysis of lung tumor initiation and progression using conditional expression of oncogenic K-ras. Genes Dev. 2001;15(24):3243–8. doi: 10.1101/gad.943001 11751630 PMC312845

[16] Madison BB, Dunbar L, Qiao XT, Braunstein K, Braunstein E, Gumucio DL. Cis elements of the villin gene control expression in restricted domains of the vertical (crypt) and horizontal (duodenum, cecum) axes of the intestine. J Biol Chem. 2002;277(36):33275–83. doi: 10.1074/jbc.M204935200 12065599

[17] Barker N, van Es JH, Kuipers J, Kujala P, van den Born M, Cozijnsen M, et al. Identification of stem cells in small intestine and colon by marker gene Lgr5. Nature. 2007;449(7165):1003–7. doi: 10.1038/nature06196 17934449

[18] Sadakata H, Okazawa H, Sato T, Supriatna Y, Ohnishi H, Kusakari S, et al. SAP-1 is a microvillus-specific protein tyrosine phosphatase that modulates intestinal tumorigenesis. Genes Cells. 2009;14(3):295–308. doi: 10.1111/j.1365-2443.2008.01270.x 19170756

[19] Murata Y, Mori M, Kotani T, Supriatna Y, Okazawa H, Kusakari S, et al. Tyrosine phosphorylation of R3 subtype receptor-type protein tyrosine phosphatases and their complex formations with Grb2 or Fyn. Genes Cells. 2010;15(5):513–24. doi: 10.1111/j.1365-2443.2010.01398.x 20398064

[20] Kotani T, Setiawan J, Konno T, Ihare N, Okamoto S, Saito Y, et al. Regulation of colonic epithelial cell homeostasis by mTORC1. Scientific Reports. 2020;10(1):13810. doi: 10.1038/s41598-020-70655-1 32796887 PMC7427982

[21] Yamashita H, Kotani T, Park JH, Murata Y, Okazawa H, Ohnishi H, et al. Role of the protein tyrosine phosphatase Shp2 in homeostasis of the intestinal epithelium. PLoS One. 2014;9(3):e92904. doi: 10.1371/journal.pone.0092904 24675817 PMC3968040

[22] Sun CX, Murata Y, Imada S, Konno T, Kotani T, Saito Y, et al. Role of Csk in intestinal epithelial barrier function and protection against colitis. Biochem Biophys Res Commun. 2018;504(1):109–14. doi: 10.1016/j.bbrc.2018.08.140 30173891

[23] Imada S, Murata Y, Kotani T, Hatano M, Sun C, Konno T, et al. Role of Src family kinases in regulation of intestinal epithelial homeostasis. Mol Cell Biol. 2016;36(22):2811–23. doi: 10.1128/MCB.00311-16 27550814 PMC5086522

[24] Park J, Kotani T, Konno T, Setiawan J, Kitamura Y, Imada S, et al. Promotion of intestinal epithelial cell turnover by commensal bacteria: Role of short-chain fatty acids. Plos One. 2016;11(5):e0156334. doi: 10.1371/journal.pone.0156334 27232601 PMC4883796

[25] McKay MM, Morrison DK. Integrating signals from RTKs to ERK/MAPK. Oncogene. 2007;26(22):3113–21. doi: 10.1038/sj.onc.1210394 17496910

[26] Feng Y, Bommer GT, Zhao J, Green M, Sands E, Zhai Y, et al. Mutant KRAS promotes hyperplasia and alters differentiation in the colon epithelium but does not expand the presumptive stem cell pool. Gastroenterology. 2011;141(3):1003–13.e1-10. doi: 10.1053/j.gastro.2011.05.007 21699772 PMC3163826

[27] Holt PR, Moss SF, Kapetanakis AM, Petrotos A, Wang S. Is Ki-67 a better proliferative marker in the colon than proliferating cell nuclear antigen? Cancer Epidemiol Biomarkers Prev. 1997;6(2):131–5. 9037564

[28] Murata Y, Kotani T, Supriatna Y, Kitamura Y, Imada S, Kawahara K, et al. Protein tyrosine phosphatase SAP-1 protects against colitis through regulation of CEACAM20 in the intestinal epithelium. Proc Natl Acad Sci U S A. 2015;112(31):E4264–71. doi: 10.1073/pnas.1510167112 26195794 PMC4534239

[29] Konno T, Kotani T, Setiawan J, Nishigaito Y, Sawada N, Imada S, et al. Role of lysophosphatidic acid in proliferation and differentiation of intestinal epithelial cells. PLoS One. 2019;14(4):e0215255. doi: 10.1371/journal.pone.0215255 31017922 PMC6481811

[30] Nusse R, Clevers H. Wnt/β-Catenin signaling, disease, and emerging therapeutic modalities. Cell. 2017;169(6):985–99. doi: 10.1016/j.cell.2017.05.016 . 28575679

[31] Barker N. Adult intestinal stem cells: critical drivers of epithelial homeostasis and regeneration. Nat Rev Mol Cell Biol. 2014;15(1):19–33. doi: 10.1038/nrm3721 24326621

[32] Scoville DH, Sato T, He XC, Li LH. Current view: Intestinal stem cells and signaling. Gastroenterology. 2008;134(3):849–64. doi: 10.1053/j.gastro.2008.01.079 18325394

