# Supplementary figures and images for: Role of Ras in regulation of intestinal epithelial cell homeostasis and crosstalk with Wnt signaling
Source: PLoS One. 2021 Aug 26;16(8):e0256774. doi: 10.1371/journal.pone.0256774 (PMC8389409; doi:10.1371/journal.pone.0256774)

A

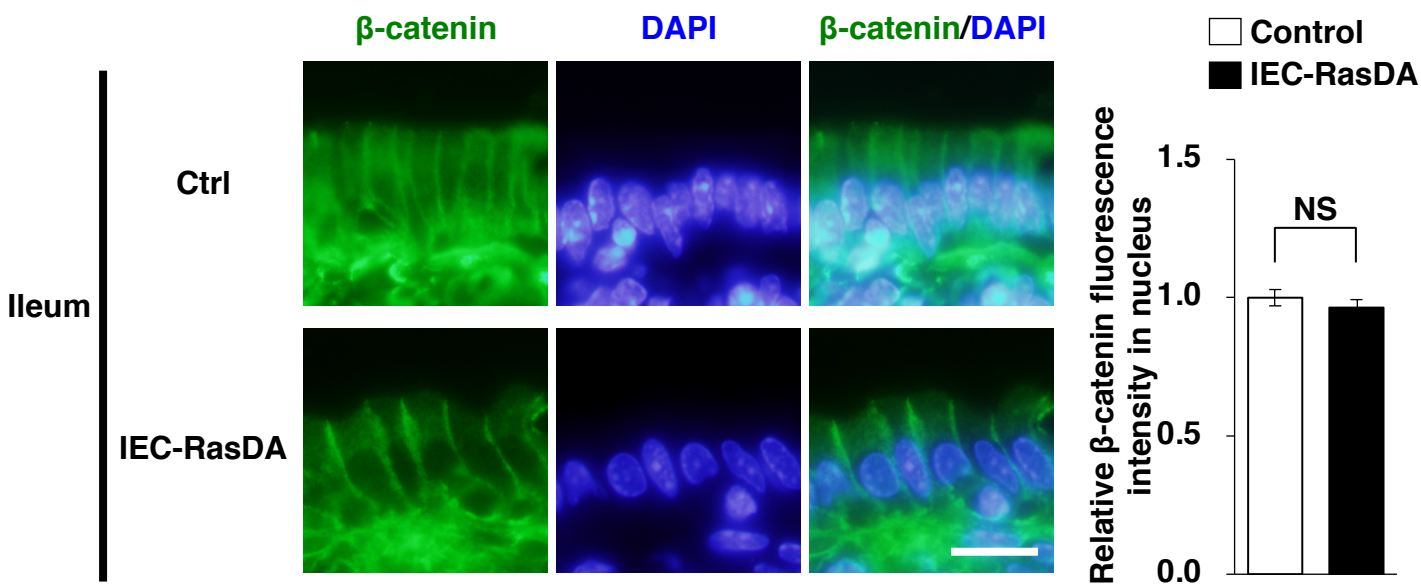

B

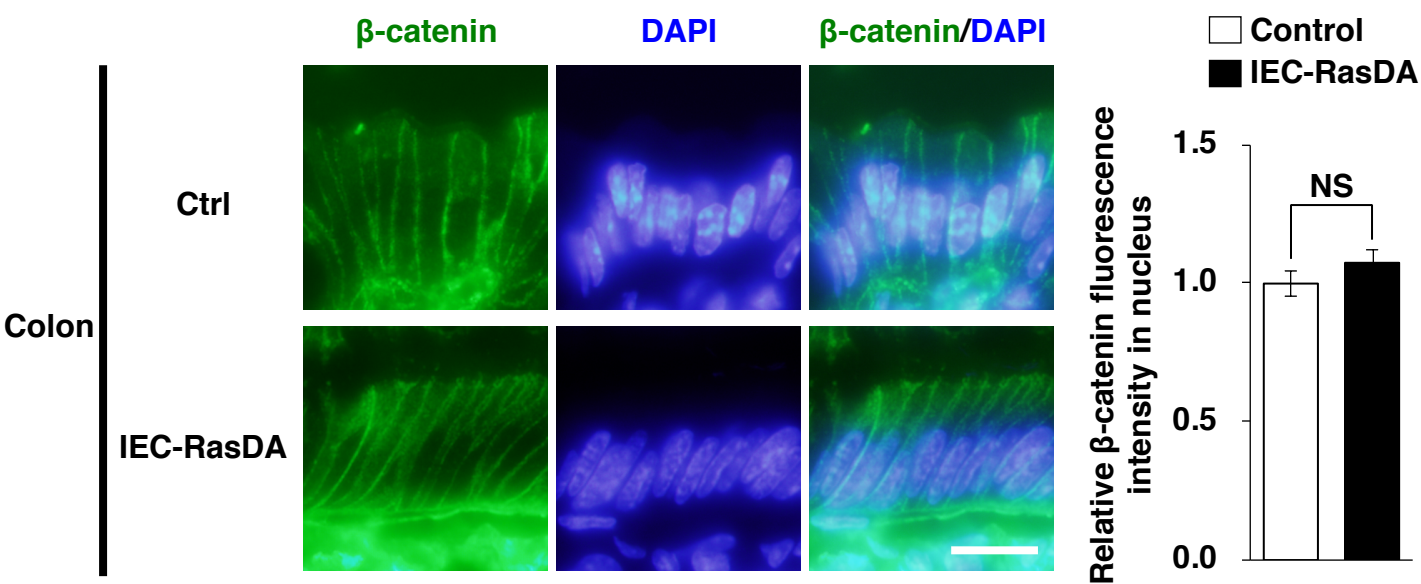

Supplement: S1 Fig — (A) Frozen sections of the ileum from control or IEC-RasDA mice were stained with antibodies to β-catenin (green) and DAPI (blue). Scale bar, 10 μm. Relative β-catenin fluorescence (green fluorescence) intensity in nucleus of IECs of the ileum is also shown in the right panel. Quantitative data are means ± s.e. for 60 IECs from control or IEC-RasDA mice. NS, not significant (Student’s t test). (B) Frozen sections of the colon from control or IEC-RasDA mice were stained with antibodies to β-catenin (green) and DAPI (blue). Scale bar, 10 μm. Relative β-catenin fluorescence (green fluorescence) intensity in nucleus of colonic epithelial cells is also shown in the right panel. Quantitative data are means ± s.e. for 60 colonic epithelial cells from control or IEC-RasDA mice. NS, not significant (Student’s t test). (PDF) [file pone.0256774.s001.pdf]

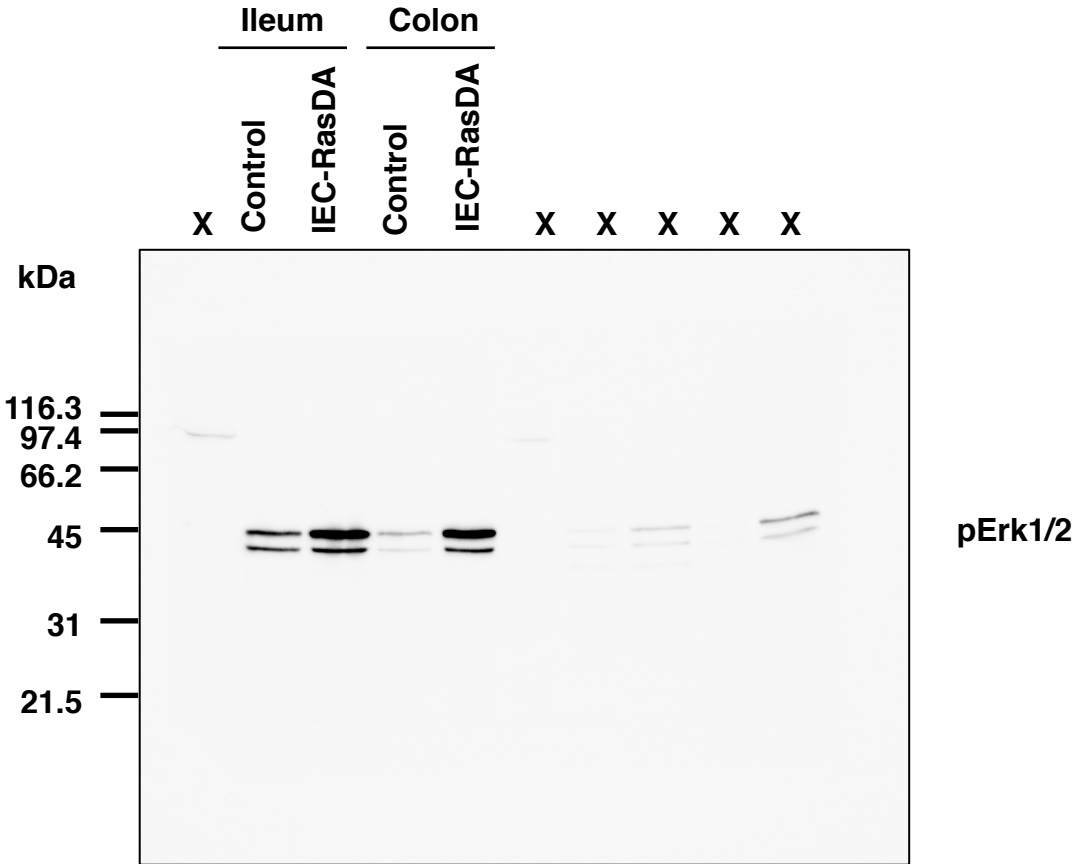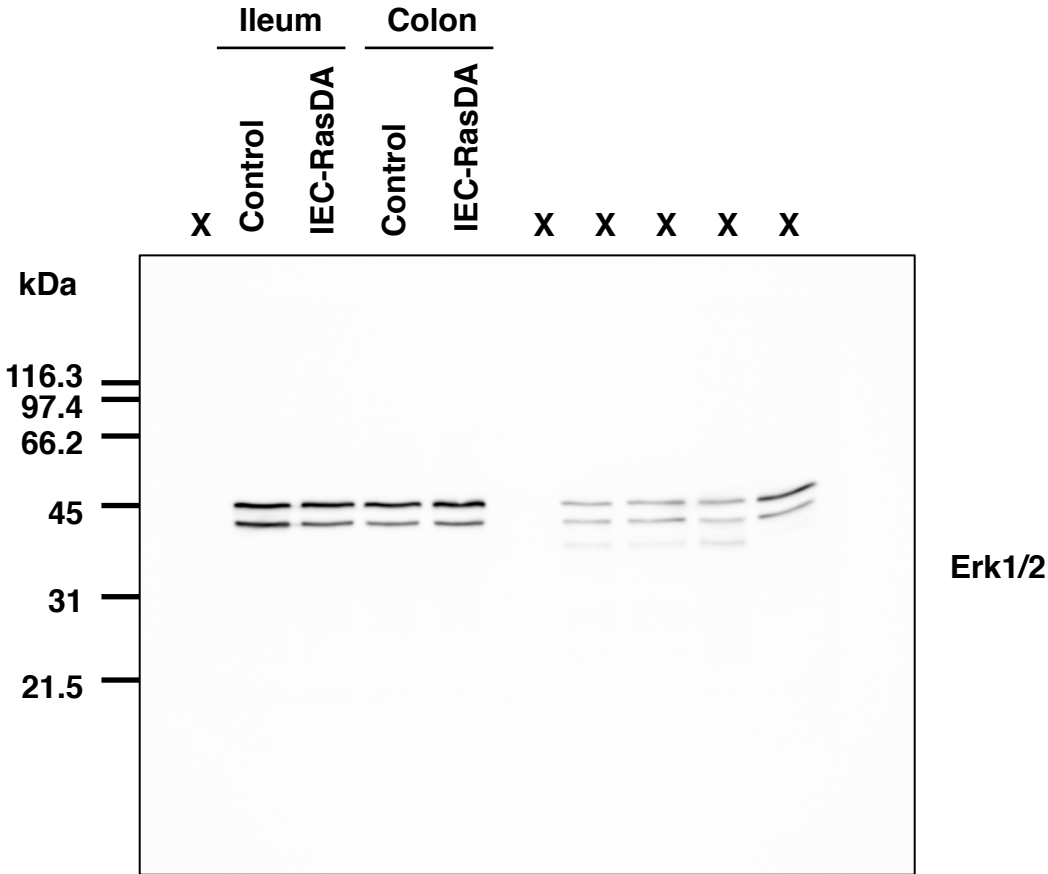

Supplement: S1 Raw images — (PDF) [file pone.0256774.s002.pdf]
